# Programmed Shape-Morphing Material Using Single-Layer 4D Printing System

**DOI:** 10.3390/mi13020243

**Published:** 2022-01-31

**Authors:** Seonjin Lee, Doyeon Bang, Jong-Oh Park, Eunpyo Choi

**Affiliations:** 1 School of Mechanical Engineering, Chonnam National University, Gwangju 61186, Korea; sj910317@naver.com; 2 Korea Institute of Medical Microrobotics, Gwangju 61011, Korea; 3 College of AI Convergence, Chonnam National University, Gwangju 61186, Korea

**Keywords:** shape-morphing, 3D printing, soft robot, microrobotics, 4D printing

## Abstract

The single-layer 4D printing technology that can be controllable in response to external stimuli is a tremendous challenge in many areas, including smart materials, robotics, and drug delivery systems. The single-layer 4D printing technique was enabled by light-focusing, which results in the difference of mechanical properties such as the coefficient of thermal expansion or Young’s modulus between focused and unfocused regions. However, 4D printing to the desired shape using single-layered material is challenging. In this paper, we demonstrate the programmed shape morphing by patterning both the static and shape-morphing layers using a single-layer 4D printing system. A shape-morphing layer is formulated by short-time (<3 s) illumination in UV light. Then a static layer is formulated by longer-time (>3 s) illumination in UV light. We expect this technique to lead to the development of micro-scale soft robots.

## 1. Introduction

The self-folding technique with soft active materials has attracted significant research in recent decades due to potential applications in medical devices and soft mechanics [1,2]. In the self-folding technique, the key is an inhomogeneous reaction. The inhomogeneous reaction occurs in an inhomogeneous membrane or bilayers of a membrane with external stimuli such as heat, electricity, and light [3,4,5,6]. The self-folding technique is a quick and reproducible production technique and can control the morphology and chemical properties of the structures [7,8]. In particular, the self-folding concept could be a practicable alternative approach for applications in the bio-medical system [1,9,10,11].

Self-folding materials consist of two layers with different properties, such as thermal expansion coefficients or Young’s modulus, affecting self-folding [4,7,10,11]. These self-folding materials fabricated by additive manufacturing can only be fabricated when the sturcture is flattenable [12]. Not every 3D shapes cannot be made by a 2D self-folding structure. To overcome this limitation, shape optimizations were developed to modify non-flattenable surfaces into flattenable [12].

However, the bilayer-based self-folding or 4D printed objects have limitations in practical application due to the structural complexness and stability of interfacial bonding between two layers [3,4].

Some research has proposed to overcome these limitations. For instance, Yoon reported a single-layer structure that can respond to temperature or pH [13]. Another single-layer structure was reported by Hao-Li, which can be shape-changing reversible through protic/aprotic stimulation [11]. In particular, they proposed a 4D printer with focused ultraviolet (UV) light, which leads to a successful single-layer self-folding structure. Also, photo-switchable actuators using liquid crystalline elastomers (LCEs), microscale object fabricated by using femtosecond laser or hydrogels were demonstrated [14,15,16,17].

**Figure 1:**
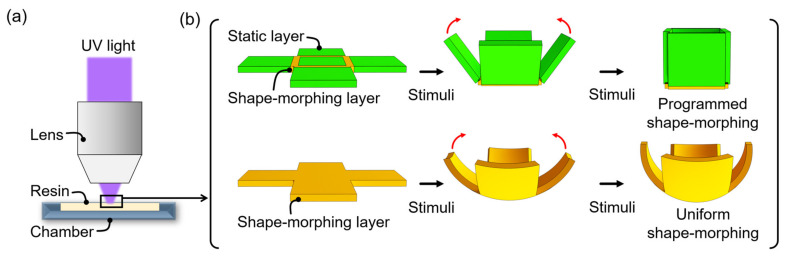
(**a**) Schematic illustration of programmed shape-morphing material using a single-layer 4D printing system. (**b**) Programmed shape-morphing (upper lane) is obtained by patterning both the static and shape-morphing layers using a single-layer 4D printing system. Uniform shape-morphing (bottom lane) is obtained when only the shape-morphing layer is used.

Although single-layer structure-based shape morphing has been successfully demonstrated, 4D printing to the desired shape using single-layered material is still challenging. Therefore, this paper demonstrates the programmed shape morphing by patterning both the static and shape-morphing layers using a single-layer 4D printing system. A shape-morphing layer is formulated by short-time (<3 s) illumination in UV light. Then the static layer is formulated by longer-time (>3 s) illumination in UV light. By using this technique, we have demonstrated the fabrication of self-folding single-layer structures with designed hinges using focused UV light (Figure 1). We selected N-isopropyl acrylamide (NIPAM) to enable shape-morphing under external stimuli, which morphs at a lower critical solution temperature (LCST) of about 33 °C. In addition, since NIPAM has good biocompatibility, we expect this technique to be applied in medical microrobots [6,18].

## 2. Experimental Section

### 2.1. Materials

All commercial chemicals were used without further purification. *N*-isopropyl acrylamide (99%, NIPAM), *N*,*N*′-methylene bis-acrylamide (99%, BIS), phenylbis (2,4,6-trimethyl benzoyl)phosphine oxide (97%, BAPO), and ethyl alcohol (99.5%, ethanol) were purchased from Sigma Aldrich (Saint Loius, MO, USA). Dimethyl sulfoxide (99%, DMSO) was purchased from Duksan (Ansan, Korea).

### 2.2. Fabrication of Photo-Curable Resin for Single-Layer 4D Printing

To obtain the NIPAM solution, 360 mg of NIPAM, 20 mg of BIS cross-linker and 20 mg of BAPO photoinitiator were dissolved in 500 μL of DMSO and 100 μL of ethanol. Then the solution was mixed at 2500 rpm for 20 min.

### 2.3. Fabrication of Programmed Shape-Morphing Materials

**Figure 2:**
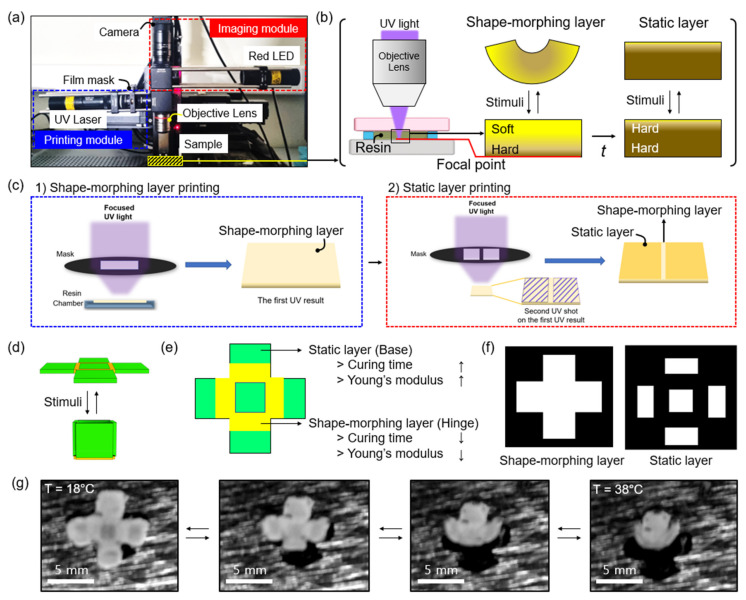
(**a**) Photograph image of the single-layer 4D printing system. (**b**) Schematic illustration of the fabrication of shape-morphing layer by using a single-layer 4D printing system. UV light emitted from UV laser is focused on the focal point (red line) using an objective lens, where the hard layer is formulated. At the same time, the soft layer is formulated in the unfocused region when UV light is emitted for a short time (<3 s). As UV light illumination time increases (>3 s), static layers form because the soft layer is turned into the hard layer. (**c**) Schematic illustration of the fabrication of programmed shape-morphing material using a two-step single-layer 4D printing process. In the first step, a shape-morphing layer is formulated by short-time (<3 s) illumination in UV light. Then static layer is formulated by longer-time (>3 s) illumination in UV light. (**d**–**f**) Programmed shape-morphing of a cross-shaped object. (**d**) Schematic illustration of programmed shape-morphing of a cross-shaped object. (**e**) Top-view illustration of the design of shape-morphing object. The object is composed of a static layer (green), where the shape does not change under stimuli and the shape-morphing layer (yellow), where the shape changes under stimuli. (**f**) Illustration of the mask for shape-morphing layer (left) and static layer (right). (**g**) Time-dependent photograph image of the cross-shaped object.

The shape-morphing object was fabricated using the optical system, as illustrated in Figure 2a. The microchamber was prepared as follows. First, 24 × 24 mm² and 20 × 20 mm² transparent slide glasses were prepared. Then a piece of double-sided tape (thickness: 150 μm; Nitto, Inc., Osaka, Japan) was attached on the slide glass to determine the resin chamber’s height. Finally, the photo-curable resin was injected directly into it via capillary force when the microchamber was ready.

For single-layer 4D printing, UV light is exposed on the micro-chamber through an objective lens (N.A.: 0.3), which results in a hard region where UV light is focused, as well as a soft region where the light is unfocused. In addition, a film mask is located between the UV laser and the objective lens to print the desired pattern.

A programmed shape-morphing material is fabricated by sequential single-layer 4D printing using different masks. In the first step, a shape-morphing layer is formulated by short-time (<3 s) illumination in UV light. Then static layer is formulated by longer-time (>3 s) illumination in UV light. Detailed mask pattern and exposure time are depicted in each figure in the manuscript.

Finally, the printed object was placed in a petri dish filled with DI water for 30 min. Then, the slide glass with tapes was removed and washed directly with additional DI water to eliminate any unpolymerized chemicals and detached with a surgical blade. Finally, the sample was placed in a petri dish with DI water.

## 3. Results and Discussion

Figure 1 describes the concept of programmed shape-morphing material using a single-layer 4D printing system. Figure 1a depicts a single-layer 4D printing system, in which a single-layered material is encoded along the longitudinal direction with an anisotropic density gradient using focused light [11]. Based on this technique, we demonstrated programmed shape-morphing objects by patterning both the static and shape-morphing layers within an object (Figure 2b). Since only the shape-morphing layer changes, whereas the static layer does not change under stimuli, programmed shape-morphing is obtained (upper lane in Figure 2b). In contrast, uniform shape-morphing is obtained when only the shape-morphing layer is used (bottom lane in Figure 2b).

Figure 2a shows the photograph image of the single-layer 4D printing system, composed of a printing module, imaging module, objective lens, and sample holder. We used a UV laser equipped with a beam-expander for the photopolymerization of an object. A film mask is located between the UV laser and an objective lens to print the desired pattern. A red light-emitting diode (LED), which has low interference with the printing module is used in the imaging module for real-time observation of the printing process. The printing and imaging modules are combined using a beam-splitter and lights are focused on the surface of the sample holder using an objective lens (Figure 2b). UV light emitted from a UV laser source is focused on the focal point (red line in Figure 2b) using an objective lens. The hard layer is formulated because the focal point receives sufficient photons for a high degree of polymerization. The soft layer is formulated in the unfocused region when UV light is emitted for a short time (<3 s; middle in Figure 2b) because the unfocused region receives insufficient photons, which results in a relatively lower degree of polymerization than the hard layer. Therefore, in this regard, both soft and hard layers coexist in a single layer, resulting in shape-morphing under stimuli. When stimuli are applied, stress is induced because the soft layer tends to shrink, whereas the hard layer still remains. This results in the folding of the printed object toward the soft layer. As UV light illumination time increases (>3 s; right in Figure 2b), static layers, consisting of only the hard layer, are formed because the soft layer is turned into the hard layer by an increasing degree of polymerization in the soft layer. The shape of the static layer does not change under stimuli because the stress is not induced by applying the stimuli.

A two-step single-layer 4D printing process is utilized (Figure 2c). In the first step, a shape-morphing layer is formulated by short-time (<3 s) illumination in UV light using a mask for the shape-morphing layer. Then, a static layer is formulated by longer-time (>3 s) illumination in UV light using a static layer mask. For example, in Figure 2c, a square-shaped shape-morphing layer is first printed (left). After that, two separated rectangles with a gap are printed as a static layer (right). This process is expected to result in a shape-morphing material that folds in half when stimuli are applied. To demonstrate the fabrication of self-morphing material using a single-layer 4D printing system, we designed a cross-shaped object as a proof-of-concept model (Figure 2d). The cross-shaped object consists of a shape-morphing layer (yellow) at the edges of the square at the center and a static layer (static layer) at the faces of squares (Figure 2e). The printed cross-shape object demonstrates Young’s modulus between static and shape-morphing layers. Young’s modulus of the static layer (base), which is exposed for 13 s, has ~10.46 MPa, while Young’s modulus of the shape-morphing layer (hinge), which is exposed for 5 s, has ~350 kPa. The fact that it is ~40 times higher in Young’s modulus of the static layer than the shape-morphing layer implies that the static layer has a higher degree of polymerization over the shape-morphing layer due to the higher photon exposure time (curing time). The static and shape-morphing layer in the cross-shaped object can be defined by using two different shape-morphing layer (left) and static layer (right) masks (Figure 2f). Using a shape-morphing layer mask and a static layer mask, a cross-shaped object with a defined shape-morphing layer and a static layer is fabricated (Figure 2g). The width of each square is ~2 mm and the thickness is ~150 μm. The width of the object can be modulated by the design of the masks and the thickness can be modulated by changing the thickness of the resin chamber (see Materials and Methods section). At 18 °C, which is below the LCST of the poly(NIPAM), the object is in a flat shape because the object is in a hydrated state. However, as the temperature increases to 38 °C, which is above the LCST of the poly(NIPAM), the shape of the object is changed to the open cube shape due to the folding of the shape-morphing layer. The dehydration of the object originates the shape-morphing. The dehydration results in the folding of the shape-morphing layer toward the soft region (see Figure 2b). The shrinkage of the soft region is steeper than the hard region due to its lower density. In contrast, the static layer’s dehydration does not result in shape-morphing because the static layer has uniform density distribution.

**Figure 3:**
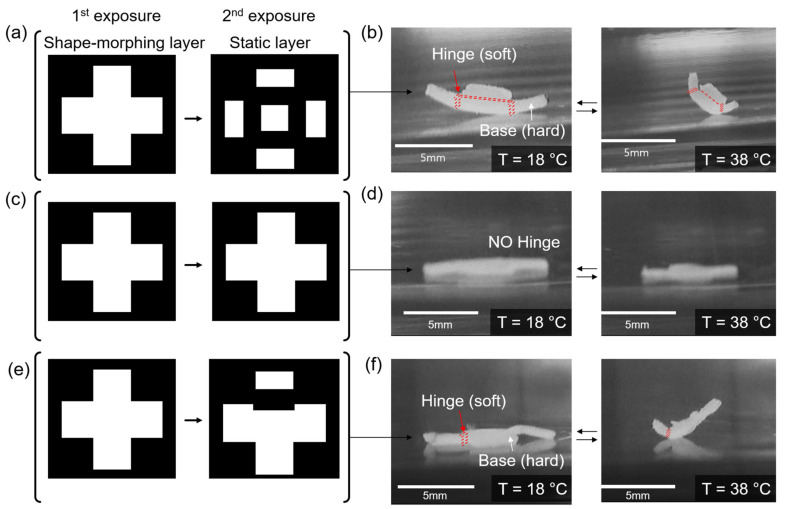
Control study of the shape-morphing material using the various masks. (**a**,**b**) Shape-morphing cross-shaped object with four hinges. Depiction of (**a**) mask pair and (**b**) photograph image of the object before (T = 18 °C) and after (T = 38 °C) shape-morphing. (**c**,**d**) Shape-morphing cross-shaped object without a hinge. Depiction of (**c**) mask pair and (**d**) photograph images of the object before and after shape-morphing. (**e**,**f**) Shape-morphing cross-shaped object a single hinge. Depiction of (**e**) mask pair and (**f**) photograph image of the object before and after shape-morphing.

We also investigated a controlled study of the shape-morphing material using the various mask pairs (Figure 3). We prepared three different mask pairs to demonstrate a cross-shaped object with four folding edges (Figure 3a,b), without any folding edges (Figure 3c,d), and with a single folding edge (Figure 3e,f). These samples are in the flat state at *T* = 18 °C because they are hydrated. However, a cross-shaped object with four folding edges folds (red line in Figure 3b) into an open cubic shape when the temperature is increased to 38 °C. In contrast, a cross-shaped object without a folding edge remains in an unfolded shape (Figure 3d). A cross-shaped object with a single folding edge demonstrates that only one face folds (Figure 3f). This result suggests that by controlling the exposure time of UV light, both shape-morphing and static layers can be achieved. In addition, by using two different mask patterns, a printed object can be programmed with defined shape-morphing and static layers.

**Figure 4:**
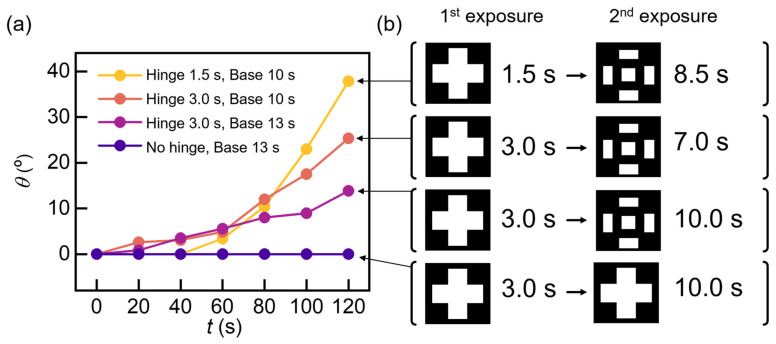
Optimization of shape-morphing. (**a**) Time-dependent folding angle (*θ*) with various first and second exposure time. (**b**) Illustration of the exposure time and the shape of the first and second exposure mask.

To optimize shape-morphing performance, we investigated the folding angle (*θ*) with respect to various UV light exposure times (Figure 4). Figure 4a depicts the time-dependent folding angle (*θ*) with various first and second exposure times, illustrated in Figure 4b. The object with 1.5 s exposure of hinge (shape-morphing layer) and 10 s exposure of base (static layer), which has the lowest UV light exposure time on hinge, exhibited the highest folding angle (~40 deg). This result suggests that the shape-morphing is caused by the induced stress between soft and hard layers after dehydration. Therefore, the highest density difference between soft and hard layers results in the highest folding angle. It is obvious that the sample with the shortest exposure time has the highest density difference between soft and hard layers. In addition, it is notable that the hinge structure is not fabricated when the sample is exposed for less than 1.5 s.

## 4. Conclusions

In summary, we demonstrated the programmed shape-morphing by patterning both the static and shape-morphing layers using a single-layer 4D printing system. A shape-morphing layer is formulated by short-time (<3 s) illumination in UV light. Then, a static layer is formulated by longer-time (>3 s) illumination in UV light. In contrast to conventional technologies, most of which require complicated fabrication or structure, our two-step single-layer 4D printing process enables the simple and rapid fabrication of programmed shape-morphing materials in the millimeter or micrometer scale. This technique is expected to enable various micro- or micro-scale soft robots by developing various stimuli-responsive materials.

## References

[1] Wei H., Zhang Q., Yao Y., Liu L., Liu Y., Leng J. (2017). Direct-Write Fabrication of 4D Active Shape-Changing Structures Based on a Shape Memory Polymer and Its Nanocomposite. ACS Appl. Mater. Interfaces.

[2] Wehner M., Truby R.L., Fitzgerald D.J., Mosadegh B., Whitesides G.M., Lewis J.A., Wood R.J. (2016). An integrated design and fabrication strategy for entirely soft, autonomous robots. Nature.

[3] Naficy S., Gately R., Gorkin R., Xin H., Spinks G.M. (2016). 4D Printing of Reversible Shape Morphing Hydrogel Structures. Macromol. Mater. Eng..

[4] Liu Y., Boyles J.K., Genzer J., Dickey M.D. (2011). Self-folding of polymer sheets using local light absorption. Soft Matter.

[5] Yuan C., Roach D.J., Dunn C.K., Mu Q., Kuang X., Yakacki C.M., Wang T.J., Yu Y., Qi H.J. (2017). 3D printed reversible shape changing soft actuators assisted by liquid crystal elastomers. Soft Matter.

[6] Stoychev G., Puretskiy N., Ionov L. (2011). Self-folding all-polymer thermoresponsive microcapsules. Soft Matter.

[7] Ionov L. (2012). Biomimetic 3D self-assembling biomicroconstructs by spontaneous deformation of thin polymer films. J. Mater. Chem..

[8] Ionov L. (2011). Soft microorigami: Self-folding polymer films. Soft Matter.

[9] Vannozzi L., Yasa I.C., Ceylan H., Menciassi A., Ricotti L., Sitti M. (2018). Self-Folded Hydrogel Tubes for Implantable Muscular Tissue Scaffolds. Macramol. Biosci..

[10] Darmawan A.D., Lee S.B., Nguyen V.D., Go G., Nguyen K.T., Lee H., Nan M., Hong A., Kim C.S., Li H. (2020). Self-folded microrobot for active drug delivery and rapid ultrasound-triggered drug release. Sens. Actuators B Chem..

[11] Li H., Darmawan A.D., Go G., Kim S.J., Nan M., Kang B., Kim H., Lee S.B., Bang D., Park J. (2021). Single-Layer 4D Printing System Using Focused Light: A Tool for Untethered Microrobot Applications. Chem. Mater..

[12] Kwok T.-H., Wang C.C.L., Deng D., Zhang Y., Chen Y. (2015). Four-Dimensional Printing for Freeform Surfaces: Design Optimization of Origami and Kirigami Structures. J. Mech. Des..

[13] Yoon C., Xiao R., Park J., Cha J., Nguyen T.D., Gracias D.H. (2014). Functional stimuli responsive hydrogel devices by self-folding. Smart Mater. Struct..

[14] Ding A., Jeon O., Tang R., Lee Y.B., Lee S.J., Alsberg E. (2021). Cell-Laden Multiple-Step and Reversible 4D Hydrogel Actuators to Mimic Dynamic Tissue Morphogenesis. Adv. Sci..

[15] Ding A., Lee S.J., Ayyagari S., Tang R., Huynh C.T., Alsberg E. (2022). 4D biofabrication via instantly generated graded hydrogel scaffolds. Bioact. Mater..

[16] Lu X., Ambulo C.P., Wang S., Rivera-Tarazona L.K., Kim H., Searles K., Ware T.H. (2021). 4D-Printing of Photoswitchable Actuators. Angew. Chem. Int. Ed. Engl..

[17] Zhang Y.L., Tian Y., Wang H., Ma Z.C., Han D.D., Niu L.G., Chen Q.D., Sun H.B. (2019). Dual-3D Femtosecond Laser Nanofabrication Enables Dynamic Actuation. ACS Nano.

[18] Go G., Nguyen V.D., Jin Z., Park J., Park S. (2018). A Thermo-Electromagnetically Actuated Microrobot for the Targeted Transport of Therapeutic Agents. Autom. Syst..

